# Supplementary material for: Automatic Grading of Individual Knee Osteoarthritis Features in Plain Radiographs Using Deep Convolutional Neural Networks
Source: Diagnostics (Basel). 2020 Nov 10;10(11):932. doi: 10.3390/diagnostics10110932 (PMC7697270; doi:10.3390/diagnostics10110932)
Supplement: Supplementary file 1 [file diagnostics-10-00932-s001.pdf]

# Automatic Grading of Individual Knee Osteoarthritis Features in Plain Radiographs using Deep Convolutional Neural Networks

**Aleksei Tiulpin**[1,2,3,*] **and Simo Saarakkala**[1,2]

[1]Research Unit of Medical Imaging, Physics and Technology, University of Oulu, Oulu, Finland.
[2]Department of Diagnostic Radiology, Oulu University Hospital, Oulu, Finland
[2]Ailean Technologies Oy, Oulu, Finland
[*]aleksei.tiulpin@oulu.fi

## Supplementary data

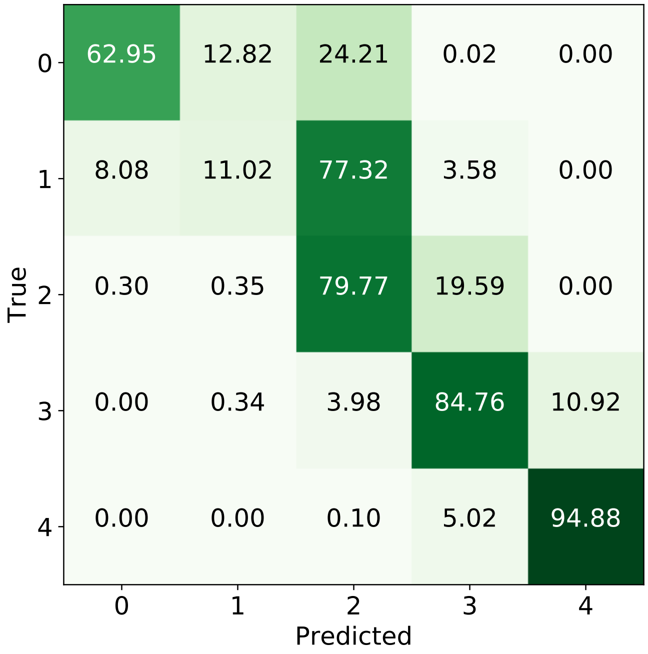


**Figure S1.** Confusion matrix for Kellgren-Lawrence (KL) grading. The numbers indicate percentages.

**Table S1.** Cross-validation results (out of fold): balanced accuracy (%) for each of the trained tasks on out-of-fold sample (OAI dataset). Best results task-wise are highlighted in bold. We selected two best models for through evaluation: SE-Resnet-50† and SE-ResNext50-32x4d‡. We trained these models from scratch (*) and also with transfer learning, but w/o the KL-grade (**). Finally, in the last row, we demonstrate the results for the ensembling of these models. L and M indicate lateral and medial compartments, FO and TO indicate femoral and tibial osteophytes and JSN indicates joint space narrowing, respectively. KL indicates the Kellgren-Lawrence grade.

| **Backbone** | **KL** | **FO** | | **TO** | | **JSN** | |
|---|---|---|---|---|---|---|---|
| | | **L** | **M** | **L** | **M** | **L** | **M** |
| Resnet-18 | 67.32 | 53.19 | 61.08 | 60.37 | 62.38 | 75.74 | 77.92 |
| Resnet-34 | 66.93 | 50.91 | 60.11 | 61.86 | 62.13 | 73.66 | 79.09 |
| Resnet-50 | 66.99 | 52.35 | 62.57 | 61.93 | 64.40 | 73.42 | 78.95 |
| SE-Resnet-50† | 67.77 | 53.96 | 62.54 | 63.22 | 65.24 | 76.13 | 78.22 |
| SE-ResNext50-32x4d‡ | 67.08 | 55.37 | 63.31 | 64.55 | 65.14 | 75.05 | 78.65 |
| SE-Resnet-50* | 65.07 | 48.58 | 54.64 | 56.14 | 56.44 | 75.86 | 78.56 |
| SE-ResNext50-32x4d* | 64.31 | 49.25 | 55.11 | 56.04 | 56.76 | 75.58 | 78.89 |
| SE-Resnet-50** | - | 54.97 | 63.28 | 64.00 | 64.17 | 73.39 | **79.65** |
| SE-ResNext50-32x4d** | - | **55.40** | 63.84 | **66.26** | 65.00 | **74.81** | 78.90 |
| Ensemble†‡ | **68.01** | 55.15 | **63.86** | 64.83 | **65.70** | **76.73** | 79.03 |

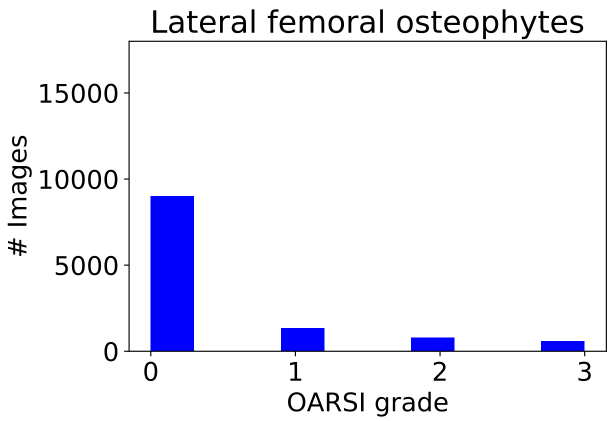


(a)

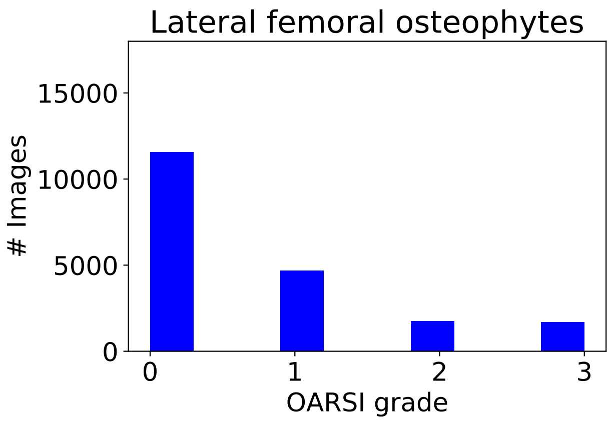


(b)

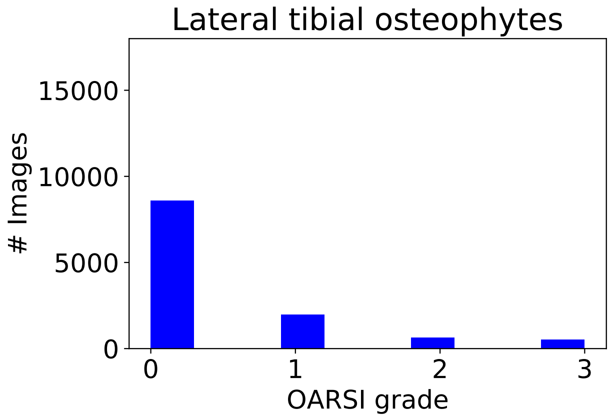


(c)

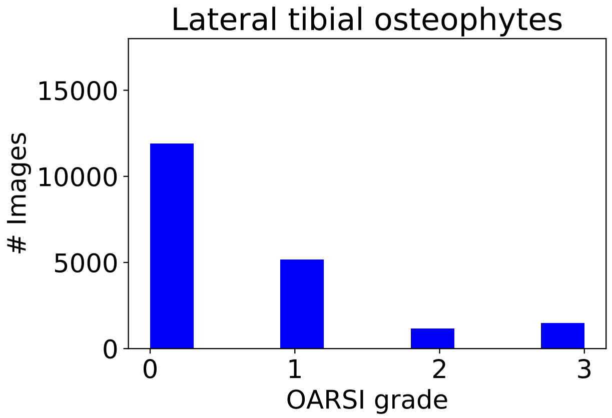


(d)

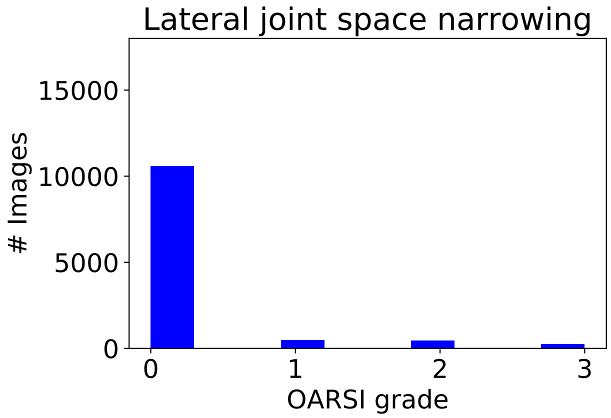


(e)

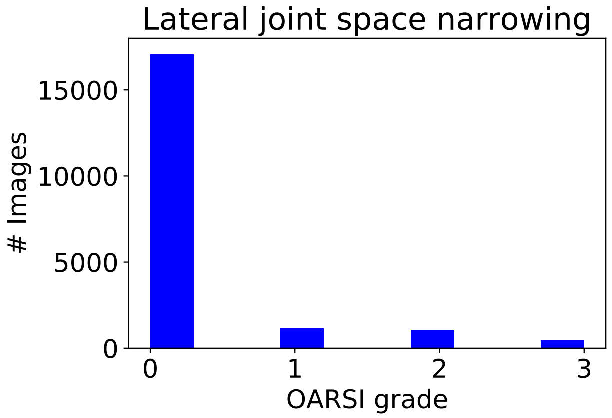


(f)

**Figure S2.** Visual representation of lateral OARSI grades distributions in MOST (S2a, S2c, S2e) and OAI (S2b, S2d, S2f) datasets.

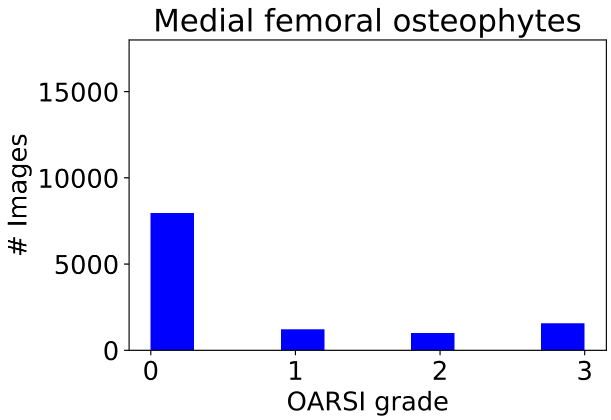


(a)

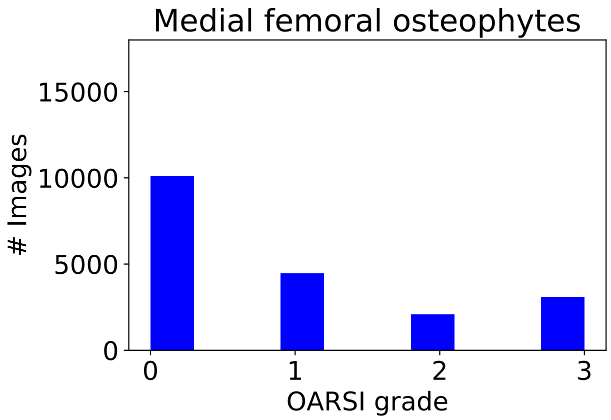


(b)

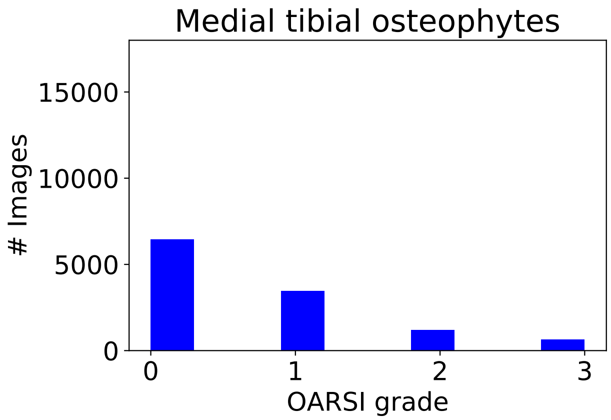


(c)

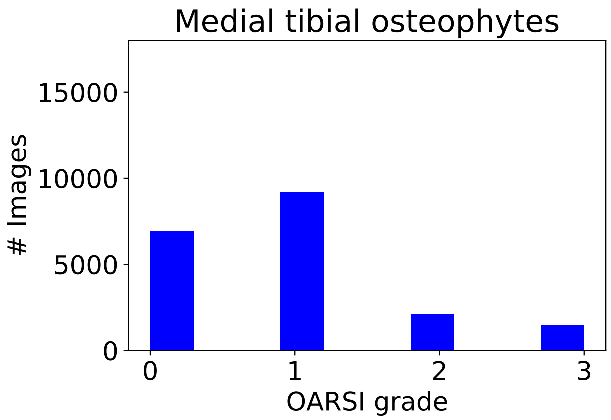


(d)

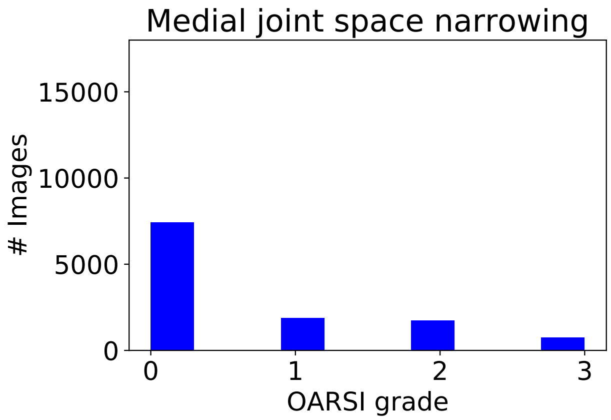


(e)

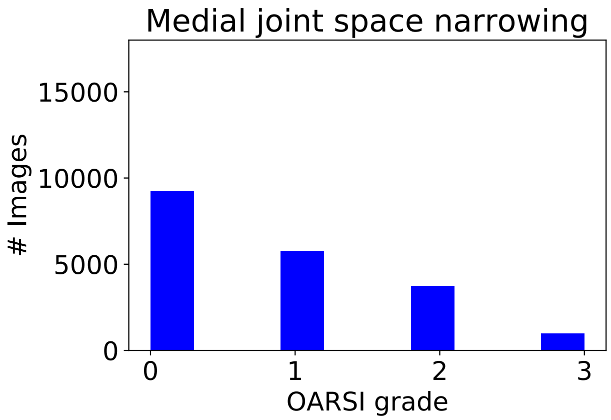


(f)

**Figure S3.** Visual representation of lateral OARSI grades distributions in MOST (S3a, S3c, S3e) and OAI (S3b, S3d, S3f) datasets.
